# Clinical characteristics, management, and outcome of tamoxifen-induced hyperlipidemia: a systematic review

**DOI:** 10.3389/fphar.2026.1838781

**Published:** 2026-09-17

**Authors:** Juan Pan, Wei Peng, MingHui Wu, Chao Ye, Xu Zhang, ZuYi Li, Xiaojuan Zhang, Qiongliang Yang, Lingzhi Zhou

**Affiliations:** 1 Department of Pharmacy, Liuyang Hospital of Traditional Chinese Medicine, Changsha, Hunan, China; 2 Department of Oncology and Hematology, Liuyang Hospital of Traditional Chinese Medicine, Changsha, Hunan, China; 3 Department of Pharmacy, Guangdong Provincial Second Hospital of Traditional Chinese Medicine (Guangdong Provincial Engineering Technology Research Institute of Traditional Chinese Medicine), Guangzhou, Guangdong, China

**Keywords:** adverse drug reaction, AP, clinical characteristics, Htg, systematic review, tamoxifen

## Abstract

**Background:**

Evidence linking tamoxifen to hypertriglyceridemia (HTG) is mainly derived from case reports. This study aimed to evaluate clinical characteristics and provide evidence to improve identification and management.

**Methods:**

Databases including China National Knowledge Infrastructure (CNKI), Wanfang, VIP, Web of Science, PubMed, Embase, and Elsevier were searched up to 25 February 2026. Case reports and case series on tamoxifen-induced hypertriglyceridemia (HTG) were collected and descriptively analyzed.

**Results:**

A total of 49 patients (2 males, 47 females) were included, with a median age of 49 years (range 34–74). Acute pancreatitis (AP) occurred in 31 patients (63.3%). Median triglyceride (TG) levels increased from 274 mg/dL before treatment to 2,534 mg/dL after tamoxifen initiation. Median time to HTG onset was 6 months, and to AP symptoms 9 months. Compared with the non-AP group, the AP group was younger (median 47 vs. 50.5 years, (p = 0.005)), had a higher prevalence of hypertension (25.6% vs. 0%, (p = 0.029)), and had higher post-treatment TG levels (3,358 vs. 1,566 mg/dL, (p = 0.051)). Logistic regression identified younger age as an independent risk factor for AP, with risk decreasing by approximately 15% per year of age (OR 0.849, 95% CI 0.750–0.961, (p = 0.01)). In three patients who underwent rechallenge, HTG recurred significantly faster (median 17.5 days). Tamoxifen was discontinued in most patients (39/49), and 81.6% received lipid-lowering therapy. TG normalization time was shorter in the AP group (median 11.5 vs. 60 days, (p = 0.003)). Forty-six patients improved. One died of liver failure, one left the hospital voluntarily due to septic shock, and one had unreported outcome. Causality was judged “probable” in 85.7% (Naranjo scale).

**Conclusion:**

Tamoxifen-induced HTG is a significant and frequent risk factor for AP, which can be severe. Younger age is inversely correlated with AP risk. Close lipid monitoring, especially during the first year of treatment and in patients with metabolic disorders, is crucial. Prompt drug discontinuation, aggressive lipid-lowering therapy, and supportive care are key to management.

## 1 Introduction

Tamoxifen (TAM), a selective estrogen receptor modulator, suppresses breast cancer cell proliferation by competitively binding to intracellular estrogen receptors, thereby antagonizing estrogen activity (Jordan, 2014). As a pioneering agent in endocrine therapy for breast cancer, TAM remains a cornerstone of treatment for both early-stage and metastatic estrogen receptor-positive (ER+/PR+) breast cancer, as recommended by the National Comprehensive Cancer Network (NCCN) guidelines (NCCN Clinical Practice Guidelines ina). It is also recommended for hormonal management of recurrent or metastatic endometrial cancer (NCCN Clinical Practice Guidelines inb). Common adverse effects associated with its anti-estrogenic properties include hot flashes, menstrual irregularities, nausea, vomiting, and edema (Yang et al., 2013). More serious adverse reactions include an increased risk of endometrial cancer, gastrointestinal malignancies, strokes and pulmonary emboli (Braithwaite et al., 2003). Notably, TAM can disrupt lipid metabolism, leading to elevated triglyceride and chylomicron levels and, in some cases, severe hypertriglyceridemia (HTG)—a condition that substantially elevates the risk of hypertriglyceridemic acute pancreatitis (AP). Since 1986, numerous case reports have documented TAM-associated HTG. Nevertheless, the incidence and clinical characteristics of this adverse effect remain poorly characterized. This study aims to elucidate the clinical features, diagnosis, and management strategies of tamoxifen-induced HTG, with the goal of providing evidence-based guidance to support its safe clinical use.

## 2 Methods

### 2.1 Search strategy

This systematic review was conducted in accordance with the Preferred Reporting Items for Systematic Reviews and Meta-Analyses (PRISMA) framework, following established methodological standards for evidence synthesis (Page et al., 2021). The following databases were searched from their inception through 25 February 2026: China National Knowledge Infrastructure (CNKI), Wanfang Data, Chinese VIP, Web of Science, PubMed, Embase, and Elsevier. No language restrictions were applied. The search strategy used the key terms (“Tamoxifen” OR “TAM”) AND (“Blood lipid levels” OR “Hyperlipidemia” OR “hypertriglyceridemia” OR “HTG”). The complete PRISMA-ScR checklist and the detailed search strategies for each database are available in Supplementary Data. The protocol for this systematic review was not registered prospectively.

### 2.2 Inclusion and exclusion criteria

Case reports and case series reporting tamoxifen-induced HTG or AP were included as preliminary study. Exclusion criteria comprised duplicate publications, review articles, mechanistic studies, animal experiments. According to the National Cholesterol Education Program Adult Treatment Panel III (NCEP ATP III) guidelines, HTG is categorized based on fasting serum triglyceride levels as follows: normal (<150 mg/dL or <1.7 mmol/L), borderline high (150–199 mg/dL or 1.7–2.3 mmol/L), high (200–499 mg/dL or 2.3–5.6 mmol/L), and very high (≥500 mg/dL or ≥5.6 mmol/L) (Expert Panel on Detection and Evaluation, 2001). According to the Atlanta classification diagnostic criteria, AP is defined by the presence of at least two of the following three features: (1) acute, persistent, typically epigastric pain that often radiates to the back; (2) serum lipase (or amylase) activity elevated to at least three times the upper limit of normal; and (3) characteristic imaging findings of AP on contrast-enhanced computed tomography (CECT), or less frequently, on magnetic resonance imaging (MRI) or transabdominal ultrasonography (Banks et al., 2013). The severity of AP was classified as follows: (1) mild AP (MAP): no organ failure and no local or systemic complications; (2) moderately severe AP (MSAP): transient organ failure (resolving within 48 h) and/or local or systemic complications in the absence of persistent organ failure; and (3) severe AP (SAP): persistent single or multiple organ failure lasting longer than 48 h (Banks et al., 2013).

### 2.3 Data extraction

Literature screening and data extraction were independently performed by two researchers according to the aforementioned criteria. The extracted information included: (1) publication source; (2) patient demographic and clinical characteristics (age, sex, primary disease, comorbidities, family history, concomitant medications); (3) tamoxifen regimen details (daily dosage and treatment duration); (4) temporal data, including date of first documented triglyceride elevation, and date of AP symptom onset; (5) laboratory and imaging findings; (6) clinical manifestations of adverse drug reactions (ADRs); (7) interventions and outcomes; and (8) causality assessment.

Using the extracted temporal data, we calculated the following intervals: latency from tamoxifen initiation to triglyceride elevation, latency from tamoxifen initiation to AP symptom onset, and (for rechallenge cases) the interval from re-exposure to recurrence. To systematically exclude alternative etiologies of AP, we extracted data on the presence or absence of common risk factors, including cholelithiasis (abdominal imaging), alcohol consumption (clinical history), hypercalcemia (laboratory data), and use of other medications known to induce AP (e.g., valproic acid, azathioprine, thiazides). All extracted data were verified and consolidated by two additional researchers, with discrepancies resolved through discussion until consensus was reached.

### 2.4 Statistical analysis

Descriptive analysis was applied to summarize patient demographics, clinical characteristics, and reported ADR profiles. A narrative synthesis approach was used to integrate the key outcomes from the included studies. Data processing and analysis were performed using SPSS version 26.0 (IBM Corporation, Armonk, NY, United States). Continuous variables were presented as medians with ranges (minimum, maximum). For normally distributed continuous variables, intergroup comparisons were conducted using the independent samples t-test. Non-normally distributed continuous variables were analyzed using the nonparametric rank-sum test. Categorical variables were expressed as frequencies and percentages (n, %), with comparisons made using the χ^2^ test or Fisher’s exact test, as appropriate. To identify independent risk factors for tamoxifen-associated HTG, variables with a *P* < 0.1 in univariate analyses were entered into a forward stepwise multivariate logistic regression model. A two-tailed *P* < 0.05 was considered statistically significant.

### 2.5 Correlation evaluation

The Naranjo Adverse Drug Reaction Probability Scale was used to assess the association between tamoxifen and HTG (Naranjo et al., 1992). Probability was assigned via a score termed definite (≥9), probable (5–8), possible (1–4), or doubtful (≤0).

## 3 Results

### 3.1 Patients’information

Following the retrieval and screening process, a total of 49 patients from 40 studies were included in this analysis (Zhang et al., 2024; Zhai et al., 2023; Li et al., 2018; Lin et al., 2020; Sánchez et al., 2005; Goraya et al., 2023; Mikhailidis et al., 1997; Colls and George, 1998; Elisaf M. S. et al., 2000; Artac et al., 2002; Brun et al., 1986; Hozumi et al., 1997; Lin et al., 2004; Alagozlu et al., 2006; Sakhri et al., 2010; Üskent et al., 2011; Czyżykowski et al., 2014; Kim et al., 2014; Noguchi et al., 1987; Singh et al., 2016; Kochar et al., 2018; Li and Khan, 2016; Sedhom et al., 2016; Wadood et al., 2016; Kataria et al., 2017; Tey et al., 2019; Jang and Kim, 2019; Yoneyama and Nakagawa, 2019; Aboutarik et al., 2020; Isobe et al., 2021; Gunay et al., 2021; Amin et al., 2021; Liljenquist, 2022; Sprague and Master, 2022; KANEL et al., 2015; Khabbal et al., 2012; Taira et al., 1998; Moussaoui et al., 2024; Jonkers, 1999; Santeufemia et al., 2009). The literature search and selection procedure is summarized in Figure 1. The study population comprised 2 males (4.1%) and 47 females (95.9%), with a median age of 49 years (range 34–74). Patients younger than 60 years accounted for 85.7% (n = 42) of the cohort. Geographically, 22 patients were from Asia (Japan, n = 7; People’s Republic of China, n = 6; Turkey, n = 4; South Korea, n = 2; India, n = 2; Singapore, n = 1), 13 from Europe (Greece, n = 8; Netherlands, n = 1; Spain, n = 1; United Kingdom, n = 1; Italy, n = 1; Poland, n = 1), 9 from North America (United States of America, n = 8; Canada, n = 1), 4 from Africa (Morocco, n = 3; Tunisia, n = 1), and 1 from Oceania (New Zealand, n = 1). All 49 patients received tamoxifen for breast cancer treatment. No alcohol use history was reported. Comorbidities were present in 31 patients. The most common conditions were hyperlipidemia (17 patients, plus 1 with a family history), type 2 diabetes (11 patients, plus 2 with a family history of diabetes), and hypertension (7 patients). Other documented comorbidities included biliary tract disease (n = 3), overweight or obesity (overweight, n = 3; obesity, n = 2), and a history of AP (n = 1). Concomitant medications were recorded for 19 patients, primarily including lipid-lowering agents, hypoglycemic drugs, and antihypertensive medications. Among the 49 patients with tamoxifen-induced HTG, 31 patients (63.3%) developed AP. Detailed clinical data are presented in Tables 1, Tables 2. Using the occurrence of AP as the dependent variable and the factors with P < 0.05 in the intergroup comparison—namely age and history of hypertension—as independent variables, logistic regression analysis was performed. As shown in Table 3, age showed a significant negative association with tamoxifen-induced hyperlipidemic AP (OR = 0.849, 95% CI: 0.750–0.961, (P = 0.01)), indicating that older age was associated with lower risk. However, the effect of hypertension could not be reliably estimated due to complete separation in the data—specifically, no patient in the non-AP group had hypertension, while seven patients (25.6%) in the AP group had this comorbidity. This resulted in an unstable OR estimate (OR = 0, P = 0.999), and the finding should be interpreted with caution.

**FIGURE 1:**
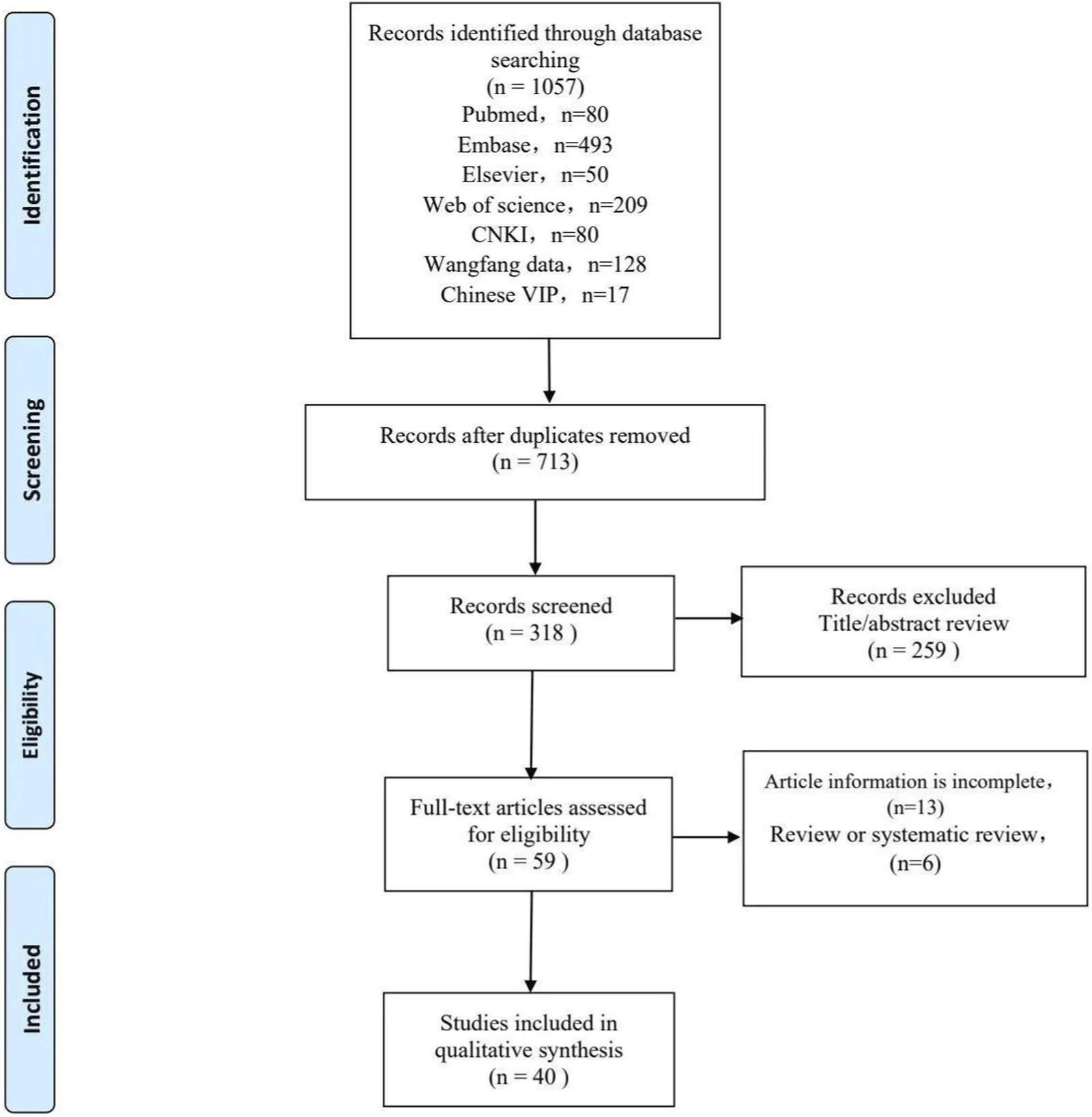
Flowchart of literature screening.

**TABLE 1:** Characteristics, clinical characteristics, treatment and outcome of the 49 included patients.

Parameter		Value
Sex (49)^a^	Male	2(4.1%)
	Female	47(95.9%)
Age (49)^a^	Years	49 (34-74)^b^
	<60 years	42(85.7%)
Region (49)^a^	The United States	8 (16.3%)
	Greece	8 (16.3%)
	Japan	7(14.3%)
	China	6(12.2%)
	Turkey	4(8.2%)
	Morocco	3(6.1%)
	South Korea	2(4.1%)
	India	2(4.1%)
	Netherlands	1(2.0%)
	Canada	1(2.0%)
	Tunisia	1(2.0%)
	Spain	1(2.0%)
	Singapore	1(2.0%)
	New Zealand	1(2.0%)
	Italy	1(2.0%)
	United Kingdom	1(2.0%)
	Poland	1(2.0%)
BMI (12)^a^	kg/m^2^	24.3(22.59-30.33)^b^
Dose of TAM (41)^a^	10 mg	2(4.9%)
	20 mg	37(90.2%)
	30 mg	1(2.4%)
	40 mg	1(2.4%)
Alcohol use		0
Accompanying diseases (31)^a^	Hyperlipidemia or family history of hyperlipidemia	18(58.1%)
	Type 2 diabetes or family history of diabetes	13(41.9%)
	Hypertension	7(22.6%)
	Biliary tract disease	3(9.7%)
	overweight	3(9.7%)
	obesity	2(6.5%)
	History of acute pancreatitis	1(3.2%)
Concomitant medications	Glimepiride,niacin,atorvastatin,gemfibrozil,acarbose,gliclazide,dulaglutide,amlodipine,cyclophosphamide, adriamycin, 5-fluorouracil,goserelin,triptorelin	19(38.8%)
Time of HTG onset(43)^a^	Months	6(0.75-60)^b^
	≤6 months	22(51.2%)
	6-12 months	7(16.3%)
	>12 months	14(32.5%)
Time of AP onset(29)^a^	Months	9(0.75-60)^b^
TAM pretreatment TG levels(23)^a^	mg/dL	274(52-1,300)^b^
TAM pretreatment TC levels(15)^a^	mg/dL	198.61(143.46-367.36)^b^
Time to hospitalization(15)^a^	Days	14(4-60)^b^
Rechallenge		3
Occurrence after rechallenge	Days	17.5(5-30)^b^
Therapy	Discontinued TAM	39(79.6%)
	Lipid-lowering medications	40(81.6%)
	Oral lipid-lowering agents alone	28(70.0%)
	Insulin combination with oral agents	9(22.5%)
	Insulin combination with low-molecular-weight heparin	2(5.0%)
	CRRT along with oral fenofibrate	1(2.5%)
	The other specific interventions	6(10.2%)^c^
	CT-guided puncture and catheter drainage placement	2(33.3%)
	Peritoneal dialysis	2(33.3%)
	Nasobiliary drainage	1(16.7%)
	Endotracheal intubation with mechanical ventilation	1(16.7%)
	Hemodialysis combined with plasma exchange	1(16.7%)
	Switched to another drug	16(32.6%)
	Letrozole	8(50%)
	Anastrozole	4(25%)
	Goserelin	1(6.2%)
	Exemestane	1(6.2%)
	Goserelin combined with exemestane	1(6.2%)
	Goserelin combined with anastrozole	1(6.2%)
Post-TAM TG Levels(31)^a^	mg/dL	2,534(221.51-20,344)^b^
Post-TAM TC Levels(31)^a^	mg/dL	435(131.48-1,800)^b^
Time to TG improvement(33)^a^	Days	16(3-300)^b^
Prognosis (49)^a^	Recover or improve	46(93.9%)
	Die	1(2.0%)
	Left the hospital voluntarily	1(2.0%)
	NA	1(2.0%)
Followed up after switching/alternative therapy(10)^a^	TG levels normalized	9(90.0%)
	TG levels elevated	1(10.0%)
Naranjo score(49)^a^	Possible	5(10.2%)
	Probable	42(85.7%)
	Definite	2(4.1%)

BMI, body mass index.NA, no data available.

^a^ Represents the number of patients out of 49 in whom information regarding this particular parameter was provided.

^b^ Median (minimum-maximum).

^c^ One patient received two interventions simultaneously.

**TABLE 2:** Comparison of Clinical Characteristics of Included Patients with or Without AP.

Parameter	Non-AP Patients(n=18)	AP Patients(n=31)	Total	Z/χ^2^ value	*P* value
Age/Years	50.5(43-74)	47(34-62)	49(34-74)	5.672	0.005
Sex/n(%)				0.158	0.691
Female	17(94.4)	30(96.8%)	47(95.9%)		
male	1(5.6)	1(3.2%)	2(4.1%)		
Underlying disease/n(%)					
Hyperlipidemia or family history of hyperlipidemia	9(50.0%)	9(29.0%)	18(36.7%)	2.154	0.142
Type 2 diabetes or family history of diabetes	5(27.8%)	8(25.8%)	13(26.5%)	0.023	0.880
Hypertension	0(0%)	7(25.6%)	7(14.3%)	4.742	0.029
Biliary tract disease	0(0%)	3(9.7%)	3(6.1%)	1.856	0.173
History of acute pancreatitis	0(0%)	1(3.2%)	1(2.0%)	0.593	0.441
Overweight or obesity	3(16.7%)	4(12.9%)	7(14.3%)	0.132	0.717
TAM pretreatment TG levels/mg/dl	283(52-1,300)	148.76(122-752.63)	274(52-1,300)	23.0	0.242
TAM pretreatment TC levels/mg/dl	244(42.54-367.36)	166.28(143.46-185.22)	198.61(143.46-367.36)	3.823	0.280
Duration of Tamoxifen Use/Months	10.5(3-60)	8(0.75-60)	6(0.75-60)	200.5	0.948
TAM Dosage/mg				154.0	0.304
10	0(0)	2(6.4%)	2(4.1%)		
20	15(83.3%)	22(71.0%)	37(75.5%)		
30	1(5.6%)	0(0)	1(2.0%)		
40	1(5.6%)	0(0)	1(2.0%)		
Not reported	1(5.6%)	7(22.6%)	8(16.3%)		
Post-TAM TG Levels/mg/dl	1566(212.51-7,200)	3358(275.4-20,344)	2,534(221.51-20,344)	172	0.051
Post-TAM TC Levels/mg/dl	363.5(174.02-1,300)	545.25(131.48-1,800)	435(131.48-1,800)	-1.492	0.161
Time to TG improvement/Days	60(7-300)	11.5(3-70)	16(3-300)	49.5	0.003
TG Recovery Level/mg/dl	218(106.25-646.38)	282(72.36-557.83)	241.24(72.36-646.38)	0.425	0.883
Recovered/improved cases/n(%)	18(100%)	28(90.3%)	46(93.9%)	1.856	0.395
Hospitalization days	7^a^	14(4-60)	14(4-60)	/	/

^a^ Hospitalization duration was reported in only one case.

**TABLE 3:** Clinical characteristics,treatment and prognosis of AP patients (n = 31).

Parameter	Clinical features	Value, n (%)
Presenting symptoms(31)^a^	Abdominal pain	26(83.9%)
	Vomiting	14(45.2%)
	Nausea	12(38.7%)
	Abdominal distension	2(6.4%)
	Fever	2(6.4%)
	Dyspnea	2(6.4%)
	Tachycardia	1(3.2%)
Severity of acute pancreatitis (31)^a^	Mild	17(54.8%)
	Moderate	11(35.5%)
	Severe	3(9.7%)
Total leukocyte count (17)^a^	Normal	7(41.2%)
	Elevated	10(58.2%)
	/mm^3^	13,600(7,700-18,500)^b^
Neutrophil percentage(17)^a^	Normal	6(35.3%)
	Elevated	11(64.7%)
	%	83.8(30-94.1)^b^
CRP levels (8)^a^	Normal	1(12.5%)
	Elevated	7(87.5%)
	mg/L	264(2.412-15000)^b^
Serum amylase levels (20)^a^	IU/L	696.5(221-1,727)^b^
Serum lipase levels (19)^a^	IU/L	1,069(114-14,258)^b^
CT (24)^a^	edema and peripancreatic collection	8(33.3%)
	necrotizing pancreatitis	5(20.8%)
	Inflammation	4(16.7%)
	Acute pancreatitis	4(16.7%)
	pseudocyst	1(4.2%)
	pancreatic enlargement	1(4.2%)
	Normal	1(4.2%)
Ultrasonogram of the abdomen (7)^a^	Pancreatic swelling	4(57.1%)
	Normal	3(42.9%)
Symptom relief time (12)^a^	Days	7(2-132)^b^
Time to pancreatic enzyme improvement (10)^a^	Days	7(3-21)^b^
Amylase level at improvement(11)^a^	IU/L	66(23-186)^b^
Lipase level at improvement(5)^a^	IU/L	75(37-233)^b^

CRP, C-reactive protein; CT, computed tomography

^a^ Represents the number of patients out of 31 total patients for whom information regarding this particular parameter was provided.

^b^ Median (minimum, maximum).

### 3.2 Administration of tamoxifen

Data on tamoxifen dosage were available for 41 cases. The daily dose of tamoxifen ranged from 10 to 40 mg, within the approved therapeutic range. Doses included 10 mg in 2 patients (4.1%), 20 mg in 37 patients (75.5%), 30 mg in 1 patient (2.0%), and 40 mg in 1 patient (2.0%). The median time to triglyceride level elevation was 6 (range: 0.75–60) months. More than half of the cases (51.2%) occurred within the first 6 months, 16.3% between 6 and 12 months, and a substantial proportion (32.5%) presented after more than 1 year of treatment. In the AP group, the median time to triglyceride elevation was 8 (range: 0.75–60) months, while the median time to symptom onset was 9 (range: 0.75–60) months. In contrast, the non-AP group had a median onset time of 10.5 (range: 3–60) months, suggesting that patients who did not develop pancreatitis had a longer latency period compared to those who developed AP. Rechallenge was performed in three patients. The median time to HTG recurrence after rechallenge was 17.5 (range: 5–30) days, which was shorter than the time to onset following the initial exposure. Details are shown in Table 1, 2.

### 3.3 Clinical presentation

The clinical manifestations of AP induced by tamoxifen-associated HTG are summarized in Tables 4. Among the 31 patients with tamoxifen-induced hypertriglyceridemic AP, abdominal pain was the most common symptom, reported in 26 patients (83.9%), followed by vomiting in 14 patients (45.2%) and nausea in 12 patients (38.7%). Other manifestations included abdominal distension, fever, and dyspnea (each reported in two patients, with one case of dyspnea progressing to acute respiratory distress syndrome), as well as tachycardia in one patient. Regarding disease severity, 17 patients (54.8%) were classified as mild AP (MAP), 11 patients (35.5%) as moderately severe AP (MSAP), and 3 patients (9.7%) as severe AP (SAP).

**TABLE 4:** Logistic regression analysis of tamoxifen-induced HTG-AP.

Risk factor	β value	Wald value	*P* value	OR value(95% CI)
Age	-0.164	6.683	0.01	0.849(0.750-0.961)
Hypertension	-21.866	0.000	0.999	0

The odds ratio for hypertension could not be reliably estimated due to complete separation. The presented OR (0) and P value (0.999) are statistical artifacts and should be interpreted with caution.

### 3.4 Laboratory tests and imaging examination

Laboratory findings for the 49 patients are summarized in Tables 2. Before tamoxifen treatment, the median TG level was 274 (range: 52–1,300) mg/dL, and the median total cholesterol (TC) level was 198.61 (range: 143.46–367.36) mg/dL. After initiating tamoxifen, one patient developed severe lipemia, precluding measurement of TG levels. Among the remaining 48 patients, the median TG level was 2,534 (range: 221.51–20,344) mg/dL. Compared with the non-AP group, the AP group exhibited higher post-TAM TG levels (1,566 mg/dL [212.51–7,200] vs. 3,358 mg/dL [275.4–20,344], p = 0.051). TC levels were recorded in 35 patients, with a median of 435 (range: 131.48–1,800) mg/dL.

Of the 31 patients who developed AP, total leukocyte counts were available for 17 cases, with elevation noted in 10 cases and a median value of 13,600 (range: 7,700–18,500)/mm^3^. Neutrophil percentage was elevated in 11 patients, with a median of 83.8 (range: 30–94.1)%. Among the eight reported patients with reported data, C-reactive protein (CRP) levels were elevated in seven patients (87.5%), with the median value of 264 (range:2.412–15,000) mg/L. The median serum amylase levels was 696.5 (range: 221–1,727) U/L in 20 patients, and the median value of serum lipase levels was 1,069 (range: 114–14,258) U/L in 19 patients. Among the 24 patients who underwent the computed tomography (CT) scans, the imaging findings included edema and peripancreatic collection in 8 cases, necrotizing pancreatitis in 5 cases, pancreatitis-associated inflammatory changes in 4 cases, and AP in another 4 cases. Additionally, one case each of pseudocyst and pancreatic enlargement was identified, while only one patient showed normal pancreatic morphology. Abdominal ultrasound examination was performed in seven patients, revealing pancreatic swelling in four patients and normal findings in three patients. Details are shown in Table 4.

### 3.5 Treatment and prognosis

The treatment and prognosis are detailed in Table 1. TAM was discontinued in 39 patients, all of whom received conventional supportive measures, including fasting, intravenous fluid resuscitation, acid suppression, enzyme inhibition, analgesia, and anti-infective therapy as clinically indicated. In addition, specific interventions were performed: peritoneal dialysis in two patients (Zhai et al., 2023), CT-guided abdominal puncture and drainage catheter in two patients (Zhai et al., 2023; Lin et al., 2020), nasobiliary drainage in one patient (Li et al., 2018), hemodialysis combined with plasma exchange in one patient (Noguchi et al., 1987), endotracheal intubation with mechanical ventilation in one patient (Sakhri et al., 2010), and continuous renal replacement therapy (CRRT) in one patient (Lin et al., 2020).

During the acute phase, 40 patients (81.63%) received lipid-lowering therapy for HTG. Among them, 28 patients were treated with oral lipid-lowering agents alone; 11 patients received insulin (9 in combination with oral agents, 2 with low-molecular-weight heparin); and 1 patient underwent CRRT along with oral fenofibrate. Following the above interventions, one critically ill patient progressed to septic shock and left the hospital voluntarily (Lin et al., 2020). Another patient, after discontinuing TAM and undergoing hemodialysis plus plasma exchange, showed a rapid decline in TG and TC levels but eventually died of liver failure (Noguchi et al., 1987). All remaining patients improved.

The median time to TG improvement was 16 (range: 3–300) days, and the median TG level was 241.24 (72.36–646.38) mg/dL. In the non-AP group, the median time to TG improvement was 60 (range: 7–300) days, with a median TG level of 218 (range: 106.25–646.38) mg/dL. In the AP group, the median time to TG improvement was 11.5 (range: 3–70) days, with a median recovery TG level of 282 (range: 72.36–557.83) mg/dL. A significant difference was observed in the time to TG improvement between the two groups, with the AP group showing a shorter time than the non-AP group (*P* = 0.003). Pancreatitis symptoms resolved after a median of 7 (range: 2–132) days, and pancreatic enzyme levels improved after a median of 7 (range: 3–21) days, with a median post-treatment amylase of 66 (range: 23–186) U/L and lipase of 75 (range: 37–233) U/L. Among the 49 patients, hospital stay was recorded in 15 patients, with a median duration of 14 (range: 4–60) days.

Alternative regimens after TAM discontinuation were documented in 16 patients. One male patient switched to goserelin combined with exemestane. Among the 15 female patients, two younger individuals (aged 36 and 40 years) switched to goserelin plus anastrozole and goserelin monotherapy, respectively. Of the remaining 13 female patients (aged 44–62 years), one 47-year-old underwent laparoscopic oophorectomy followed by exemestane; the others received letrozole (n = 8) or anastrozole (n = 4). Follow-up after switching was recorded in 10 patients. In one case (Jang and Kim, 2019), serum TG levels rose again after switching to letrozole but returned to normal after adding fenofibrate. The remaining patients tolerated the alternative regimens well, with no recurrence of elevated serum TG levels.

### 3.6 Relevance evaluation

Of all 49 cases, 42 patients (85.71%) were assessed as having probable tamoxifen-induced HTG according to the Naranjo algorithm. Five patients (10.21%) were classified as possible cases (score of four in four patients, score of three in one patient). Two patients (4.08%) were rated as definite, both with a score of 9.

## 4 Discussion

Among the 49 patients with tamoxifen-associated HTG, 85.7% were under 60 years of age. Notably, age was inversely correlated with the risk of TAM induced HTG-associated AP (HTG-AP), as patients who developed AP were significantly younger than those who did not. Multivariate logistic regression analysis further confirmed that younger age is an independent risk factor for TAM-induced HTG-associated AP. This phenomenon may be explained by the fact that the aging pancreas exerts inhibitory regulation on acinar cell secretory function via miR-503-322 derived from pancreatic islet β-cells, whereas younger individuals lack this regulatory mechanism, leading to more active pancreatic function and an exaggerated response to pathogenic stimuli (Liu et al., 2025). The cohort was predominantly female (95.9%), consistent with the epidemiology of breast cancer, in which male cases constitute fewer than 1% of all diagnoses (Si et al., 2022). Nevertheless, the occurrence of this adverse event in male patients suggests that the risk is not sex-specific. Clinicians managing male breast cancer patients on endocrine therapy should remain equally attentive to this potential complication.

The onset of tamoxifen-associated HTG was highly variable, occurring from 3 weeks to 5 years after treatment initiation, with a median onset time of 6 months—a finding consistent with prior studies (Bhunisha et al., 2023). Notably, the median time to the development of AP was 9 months post-tamoxifen initiation. This discernible 3-month interval between the median detection of significant HTG and the subsequent clinical manifestation of AP defines a critical window for timely intervention. This temporal pattern strongly underscores the necessity of long-term metabolic monitoring, emphasizing that the risk extends significantly beyond the initial treatment phase.

A key finding of this study is that 63.3% of patients with tamoxifen-induced HTG developed AP. This indicates that HTG in this patient population is not merely an incidental biochemical abnormality, but rather a critical warning sign for progression to a life-threatening complication. Further analysis revealed that although the total duration of tamoxifen exposure and mean triglyceride levels did not differ significantly between the AP and non-AP groups, the AP group exhibited two distinct characteristics: earlier onset of HTG (median: 8 months vs. 10.5 months) and higher peak triglyceride levels at the time of HTG diagnosis (median: 3,358 mg/dL vs. 1,566 mg/dL). This study demonstrates that both the absolute level and the rate of increase of triglycerides are important predictors of AP risk in patients receiving tamoxifen therapy. On one hand, consistent with previous reports (Pedersen et al., 2016), serum TG levels are associated with AP risk in a dose-dependent manner. On the other hand, our findings reveal that, even with comparable cumulative drug exposure, a rapid and pronounced elevation of TG early in the treatment course is significantly associated with the development of AP. This highlights the clinical importance of dynamic monitoring of TG trends, particularly during the initial phase of tamoxifen therapy, as it may be more effective than single time-point measurements in identifying individuals at high risk for AP.

The present study demonstrates that tamoxifen-associated HTG can occur even in patients with previously normal lipid profiles, with triglyceride levels rising to life-threatening extremes exceeding 20,000 mg/dL. This finding contrasts with previous reports suggesting that tamoxifen significantly elevates triglycerides primarily in individuals with pre-existing disorders of triglyceride-rich lipoprotein metabolism (Elisaf M. et al., 2000). Our data indicate that the metabolic impact of tamoxifen may be more pervasive and unpredictable than previously recognized, warranting universal lipid monitoring regardless of baseline risk status. The marked inter-individual variability in post-tamoxifen triglyceride levels—ranging from 221.51 mg/dL to over 20,000 mg/dL—further underscores the role of personal susceptibility, which may be explained through several intersecting mechanisms. First, at the pharmacogenetic level, polymorphisms in drug-metabolizing enzymes such as CYP2D6 can alter the metabolic activation of tamoxifen, thereby modulating its downstream effects on lipid homeostasis (Zhou et al., 2022). Second, from a pathophysiological perspective, patients with pre-existing HTG may have near-saturated triglyceride clearance pathways, rendering them exquisitely sensitive to the triglyceride-elevating effects of tamoxifen (Kataria et al., 2017). Third, clinically, the presence of underlying metabolic disorders appears to be a critical predisposing factor (Goraya et al., 2023). In our study, most patients had comorbidities such as dyslipidemia, type 2 diabetes, or a relevant family history. Strikingly, hypertension was significantly more prevalent in the AP group (25.6% vs. 0%, P = 0.029), suggesting it may serve as a clinically useful predictor for identifying patients at higher risk of progressing to AP.

Almost half of the patients with AP developed moderately severe or severe disease, with complications including acute respiratory distress syndrome and acute kidney injury, indicating a tendency toward severe clinical progression. This finding contrasts with previous reports, which characterized drug-induced hypertriglyceridemic pancreatitis as predominantly mild or moderate in severity (Elkhouly et al., 2019). Notably, although the final triglyceride recovery levels were comparable between groups, the time to triglyceride improvement was significantly shorter in the AP group than in the non-AP group (median: 11.5 days vs. 60 days, *P* = 0.003). This difference may be attributed to the prompt clinical interventions triggered by the occurrence of AP—including drug discontinuation, intensive lipid-lowering therapy, and fasting—whereas HTG in non-AP patients, lacking such a clinical warning signal, received relatively delayed management. This phenomenon suggests that severe complications, to some extent, “catalyze” the treatment process, further underscoring the clinical importance of early identification and proactive intervention for asymptomatic HTG.

Upon diagnosis of tamoxifen-related HTG, discontinuation of the drug is recommended. For patients who have progressed to HTG-AP, comprehensive supportive management—including fluid resuscitation, nutritional support, acid suppression, enzyme inhibition, and analgesia—should be initiated promptly under close vital sign monitoring. Importantly, persistently elevated TG levels during the early phase of HTG-AP lead to excessive free fatty acid release, directly exacerbating pancreatic injury. Therefore, achieving early TG reduction to below 500 mg/dL represents a critical therapeutic target to interrupt this pathological cascade (Valdivielso et al., 2014). Current lipid-lowering approaches in clinical practice include insulin therapy, plasmapheresis, and heparin treatment (IAP/APA/EPC/IPC/JPS Working Group, 2025). However, our study found that nearly one-fifth of HTG-AP patients did not receive standardized lipid-lowering therapy during the acute phase, and the clinical lipid-lowering regimens varied widely. This situation largely stems from the current lack of high-level, specific recommendations in both domestic and international AP management guidelines regarding acute-phase lipid control in HTG-AP. Thus, further high-quality studies are urgently needed to establish clear clinical guidance for lipid-lowering therapy. Furthermore, rechallenge data in this study indicate that re-exposure to tamoxifen carries a risk of recurrent HTG; therefore, rechallenge should be avoided and alternative endocrine therapies considered.

Although a prospective multicenter randomized trial (Shien et al., 2018) reported no significant change in serum TG levels in patients treated with toremifene compared to baseline, recent case reports have documented severe HTG and AP induced by toremifene (Lv and Shen, 2022), highlighting the need for careful evaluation when selecting alternative agents. Regarding alternative treatment options, this study included goserelin, letrozole, anastrozole, and exemestane. It should be noted that aromatase inhibitors also carry a risk of dyslipidemia. Steroidal aromatase inhibitors (e.g., exemestane) generally have a milder impact on lipid profiles compared to non-steroidal agents (e.g., letrozole, anastrozole) and are therefore preferred in patients with preexisting dyslipidemia (Wang et al., 2020). This suggests that steroidal aromatase inhibitors may offer an advantage in the selection of alternative therapies. Importantly, regardless of the alternative regimen chosen, regular follow-up of lipid levels remains essential to monitor and prevent dyslipidemia-related adverse effects.

TAM competitively binds to estrogen receptors, disrupting the normal physiological regulation of lipid metabolism. This leads to a decrease in cholesterol and low-density lipoprotein cholesterol levels, while simultaneously elevating triglyceride (TG) levels (Bhunisha et al., 2023; Cheung et al., 2019). The severe HTG triggered by TAM is likely associated with altered lipid metabolism mediated by estrogen receptor modulation and abnormal hepatic lipid metabolism. Specifically, TAM reduces the activity of lipoprotein lipase (LPL) and hepatic triglyceride lipase (HTGL), thereby inhibiting TG metabolism (Hozumi et al., 1998). This finding is supported by the lipoprotein fraction analysis results obtained from three patients in our study (Brun et al., 1986; Isobe et al., 2021; Taira et al., 1998). Furthermore, long-term use of TAM may induce metabolism-associated fatty liver disease, resulting in the accumulation of TG within hepatocytes and subsequently impairing the liver’s capacity to metabolize circulating TG (Chen et al., 2018). However, the precise molecular mechanisms remain unclear.

Several limitations of this study should be acknowledged. First, this retrospective study based on published case reports was not prospectively registered, which represents a methodological limitation. Publication bias is also inherent to this design, as severe or atypical cases are more likely to be reported. Second, the included case reports showed substantial heterogeneity in data quality, monitoring frequency, diagnostic criteria, and follow-up duration, which may affect the reliability of our findings. Third, the relatively small sample size restricted statistical power for multivariate analyses and refined subgroup stratification. Fourth, the assessment of time to TG normalization was limited by the lack of standardized monitoring schedules; we uniformly used the longest follow-up TG value as the endpoint to maintain consistency, but variability in monitoring frequency may have introduced bias. Thus, prospective cohort studies with larger samples and standardized protocols are needed to confirm these findings and optimize management strategies.

## 5 Conclusion

This analysis establishes tamoxifen-induced HTG as a clinically significant adverse event, with over 60% of affected patients progressing to AP. To mitigate this risk, routine assessment of fasting lipid profiles is recommended prior to initiating tamoxifen therapy, particularly in high-risk individuals with pre-existing metabolic conditions such as dyslipidemia or diabetes. Frequent monitoring should be maintained during the early treatment phase (especially the first 6–12 months) to enable timely detection of lipid abnormalities. Upon identification of TG > 500 mg/dL, prompt initiation of lipid-lowering therapy and evaluation for tamoxifen discontinuation are warranted to prevent the development of AP. Furthermore, patient education on warning symptoms—including abdominal pain, nausea, and vomiting—should be emphasized to facilitate early recognition and prompt medical evaluation.

## Data Availability

The raw data supporting the conclusions of this article will be made available by the authors, without undue reservation.
